# Locally advanced cervical cancer: Neoadjuvant chemotherapy plus radical surgery an alternative approach to chemo-radiation in a low-income setting: A descriptive study

**DOI:** 10.1371/journal.pone.0310457

**Published:** 2024-10-30

**Authors:** Malede Birara, Tadesse Urgie, Abraham Fessehaye Sium

**Affiliations:** Department of Obstetrics and Gynecology, St. Paul’s Hospital Millennium Medical College, Addis Ababa, Ethiopia; University of Nigeria Teaching Hospital, NIGERIA

## Abstract

**Objective:**

To describe the treatment outcomes of locally advanced cervical cancer managed with neoadjuvant chemotherapy (NACT) plus radical surgery at a gynecology oncology center in Ethiopia.

**Methods:**

This was a retrospective descriptive study of management of locally advanced cervical cancer (LACC) at St. Paul’s Hospital Millennium Medical College (Ethiopia) over 5 years. Data were collected by reviewing patient records. Data were analyzed using SPSS version 23. Simple descriptive analysis was employed to analyze clinical, histologic, and treatment outcomes of LACC managed with NACT+ radical surgery. Frequency and proportions were used to present the results’ significance.

**Results:**

A total of 98 patients were analyzed. One-third (31.6%) of cervical cancer patients with locally advanced disease were operable after neoadjuvant chemotherapy. Out of this, nodal metastasis was found in 2 patients (all pelvic lymph node metastasis). Disease recurrence within 2 years was 3% (1 recurrence within 6–12 months and 2 recurrences at 12–24 months).

**Conclusion:**

This study supports utilization of NACT plus radical surgery for locally advanced cervical cancer, where chemoradiation is not readily available. Our findings imply that this treatment modality is a life-saving alternative treatment in a low-income setting, which is often married by shortage or unavailability of radiotherapy at the needed time before disease progression ensues.

## 1.Introduction

According to recent estimates, cervical cancer is the fourth most common cancer in women, ranking after breast cancer, colorectal cancer and lung cancer. Approximately, there were 570 000 cases of cervical cancer and 311 000 deaths from the disease in 2018. It was the leading cause of cancer-related death in women in eastern, western, middle, and southern Africa during that time [1]. External pelvic radiation and brachytherapy has been the standard therapy for cervical cancer patients with advanced disease stage, for which survival is guarded (5-year survival rate is only 60–70%) [2,3]. This poor prognosis is due to lymph node metastasis, vascular space invasion, undiagnosed parametrial extension, deep cervical invasion and radioresistant hypoxic tumor centers associated with advanced disease stage [3]. In recent times, additional treatment options (three-modality treatment: radiotherapy, chemotherapy, and surgery) have been investigated for the treatment of cervical cancer with the aim of increasing the efficacy of radiotherapy [4].

Defined as local disease that extends beyond that can be treated with surgery alone, locally advanced cervical cancer is a typical presentation of unscreened women [5]. Currently, there is level-A evidence that shows that the combination of radiotherapy and chemotherapy is superior for treatment of locally advanced cervical cancer (LACC) than radiotherapy alone with similar adverse effects profile between both approaches [6]. Although this combination of radiotherapy and chemotherapy is a well-studied and effective management for advanced cervical cancer cases, unavailability or difficulty in accessing radiotherapy precludes it from being a standard of care in low-income settings, such as the Sub-Saharan Africa. In our Hospital, St. Paul’s Hospital Millennium Medical College (in Ethiopia), neoadjuvant chemotherapy (NACT) followed by radical surgery has been introduced as an alternative intervention to address cervical cancer disease progression and mortality associated with delays in accessing radiotherapy (until recently there had been only one radiotherapy center in Ethiopia). However, there is limited evidence on the utility of NACT followed by surgery as an alternative treatment modality for patients with LACC. The available evidence on this topic is based on too few studies (exclusively done in high-income settings). Our study presents one of the first information on this topic from low-income setting. It describes a treatment modality of combination of NACT and radical surgery for cervical cancer patients with locally advanced disease at a gynecology oncology treatment center in Ethiopia.

## 2.Methods and materials

### 2.1 Study design, study setting, and study period

This study is a retrospective descriptive analysis of locally advanced cervical cancer cases that were treated with neoadjuvant chemotherapy plus radical surgery at St. Paul’s Hospital Millennium Medical College in Ethiopia over five years (January 2017 to December 2021. We conducted this study from September 15, 2023, to November 30, 2023. St. Paul’s Hospital is among the few tertiary hospitals offering advanced gynecologic oncology sub-specialty care and fellowship training in Ethiopia. The practice of administering neoadjuvant chemotherapy followed by radical surgery for locally advanced cervical cancer cases has been in place for the past six years, shortly after the launch of the gynecology oncology fellowship program in 2016. This intervention has remained a valuable alternative to the standard management of cervical cancer, as radiotherapy has been inaccessible for most patients due to long waiting lists. The primary outcome of the study was the operability of the tumor after neoadjuvant chemotherapy and disease recurrence within two years post-operatively.

### 2.2 Study population and procedures

The study involved a comprehensive review of records of cervical cancer patients with locally advanced disease who received neoadjuvant chemotherapy (NACT) with the intent of achieving feasible radical surgery following NACT. Clinical staging for these patients was determined using the FIGO 2009 staging system, although some cases after 2018 were assessed using the FIGO 2018 staging system. For those who could afford it, either a CT or MRI imaging was performed, and histological confirmation was obtained for all participants. The chemotherapy treatment protocol involved assessing the patient’s performance status using ECOG criteria (less than or equal to 2) and ensuring normal laboratory results for complete blood count (CBC), liver function test(LFT), and renal function test (RFT). The chemotherapy regimen consisted of paclitaxel at a dose of 175 mg/m2, administered via infusion within 3 hours, followed by carboplatin at a dose calculated based on an area under the curve of 6 (with a maximum dose of 750 mg) over 30 minutes. Pre-medications included 50 mg of I.V. diphenhydramine 30 to 60 minutes before paclitaxel, 50 mg of I.V. ranitidine 30 to 60 minutes before paclitaxel. Ondansetron 8mg, intravenously and Dexamethasone at 20 mg P.O was also administered. Antiemetics were also provided for 3 days after chemotherapy. The chemotherapy was repeated every 21 days, and on each cycle, toxicity was assessed by updating CBC, LFT, and RFT, with dose adjustments made accordingly.

After three cycles of chemotherapy, patients were reevaluated under general anesthesia to assess their response and operability. Down staging was defined by a tumor size of less than 4 cm and an absence of parametrical infiltration for stage IIB patients, as determined by a gynecologic oncologist. The treatment response for all patients was evaluated using RECIST criteria, which classifies responses as complete response, partial response, stable disease, or progressive disease. Those with at least a partial response underwent type C radical hysterectomy and pelvic lymph node dissection, following Querlow and Morrow classification guidelines. Patients with stable or progressive disease were referred for chemo-radiation. In this study Nine patients who were found to have bladder involvement after laparotomy were referred for chemo-radiation, bypassing surgery. Following surgery, pathological results were reported using TNM criteria, and those meeting the PETER’s high-risk criteria were referred for chemo-radiation.

Patients were followed for 2 to 5 years, with appointments every 3 months that included history-taking, physical examinations, and pelvic examinations. Depending on the clinical condition, vaginal smears and advanced imaging were ordered, and if recurrence was detected, biopsies were taken, and the information was recorded in the patient’s file. If a patient passed away in the hospital, it was noted in the patient’s chart.

### 2.3 Data collection

Data on clinical characteristics and prognosis, including disease stage, histologic type, chemotherapy regimen, extent of radical surgery, and survival analysis, were collected by reviewing medical records and pathologic examination reports. A structured data extraction form was employed for data extraction. Data collection was carried out from September 15, 2023- November 15, 2023, after Ethical clearance was obtained. Exclusion criteria for the study were incomplete data and patients who initiated treatment at other hospitals before being referred for further management at our hospital. Secondary outcomes focused on intra-operative and post-operative complications.

### 2.4 Sample size and sampling procedure

There was no specific sample size calculation for this study; all cases meeting the inclusion and exclusion criteria were included. Data analysis was performed using SPSS version 23software packages, with simple descriptive analysis applied to the data, presenting results in percentages and frequencies.

### 2.5 Ethical clearance

Ethical approval was obtained from the Institutional Review Board (IRB) at St. Paul’s Hospital Millennium Medical College. Informed consent from study subjects was not required according to the ethical clearance, and patients’ confidentiality was diligently maintained throughout the data collection process.

## 3. Results

In this study, a total of 98 patients with locally advanced cervical cancer were included. The mean age of the study patients was 49.1(+/-10.8) years (Table 1). The majority of them were parous (98%) and had address outside Addis Ababa city (place where the study setting is found). By far vaginal bleeding was the most common presenting complaint, represented in 96 of the patients (98%), followed by lower abdominal pain experienced by 41 patients (41.8%). Thirty-four patients (34.7%) had weight loss. More half (59.2%, 58/98) were HIV sero-negative while 11 patients were HIV-positive patients. Close to two-third (64.3%, 63/98) were at ≤Stage-IIB, from this 25.5% (25/98) were stage-IIB, before starting the neoadjuvant chemotherapy.

**Table 1:** Demographic and clinical characteristics of locally advanced cervical cancer in Ethiopia, 2017-2022(n = 98).

Variable	Category	n	%
Age (Years)	Mean	49.1(+/-10.8)	
Parity	Nulliparous	2	2.0
	Parous	96	98.0
Address	Addis Ababa	38	38.8
	Out of Addis	60	61.2
Had lower abdominal pain?	No	57	58.2
	Yes	41	41.8
Had vaginal bleeding?	No	2	2.0
	Yes	96	98.0
Had weight loss?	No	64	65.3
	Yes	34	34.7
Had urinary symptoms?	No	91	92.9
	Yes	7	7.1
HIV sero-status?	No	58	59.2
	Positive	11	11.2
	Unknown	29	29.6
Disease stage when NACT was started	1B1-1B3	27	27.6
	2A1-2A2	38	38.8
	2B	25	25.5
	3A-4A	8	8.1

The commonest histology diagnosis was keratinizing Squamous cell carcinoma (KSCC)/ non-keratinizing Squamous cell carcinoma (NKSCC), accounting for 86.7% (85 out of the 98 patients had this histologic type). Adeno carcinoma diagnosed in 10 patients (10.2%) and clear cell carcinoma in one patient were the other histologic types of cervical cancer among the patients included in this study (Table 2). After the initiation of NACT 13 patients disappeared and the rest finished the three courses of chemotherapy. Clinical response was assessed by RECIST criteria using CT and MRI and examination under anesthesia (EUA.) Eight patients showed disease progression to stage III to IVA and were primarily referred to chemo radiation. Hence, 75 cases were reevaluated under anesthesia. Among these, 44 (44.9% out of the total 98) patients showed stable disease with tumor size greater than 4 cm and parametrical involvement, hence they were all referred to chemo radiation. The remaining 31 (31.6% out of the total 98) patients showed partial response with tumor size less than 4 cm and found to be operable. A total of 3 (3%) patients had disease recurrence within 2 years (one patient within 6 months and 2 patients at 1–2 years). There was no death at 2 years’ follow-up.

**Table 2:** Histologic types and disease progress after NACT treatment for a locally advanced cervical cancer in Ethiopia, 2017-2022(n = 98).

Variable	Category	n	%
Histologic type	KSCC/NKSCC	85	86.7
	Adeno Carcinoma	10	10.2
	Clear Cell Carcinoma	1	1.0
	Other	2	2.0
	No	0	0.0
Disease progress and operability after NACT	Partial response(operable)	31	31.6
	Stable disease (Declared in-operable after EUA and referred for chemoradiation)	44	44.9
	Progressive disease (sent directly for primary chemoradiation without EUA)	8	8.2
	Disappeared	13	13.3
Recurrence in 6–12 months	Unknown	18	18.4
	No	79	80.6
	yes	1	1.0
Recurrence in 12–24 months	Unknown	18	18.4
	No	78	79.6
	yes	2	2.0
	Unknown	18	19

For the operable cases Type C Radical hysterectomy with pelvic lymph node dissection was done (Table 3). The overall intra-operative complication was 16.1% (2 bowel injury, 3 hemorrhage requiring blood transfusion, and one vascular injury). A total of 7(22.6%) patients had post-operative complications (2 wound infection, 4 DVT, and one PTE). The post-operative pathology report showed that 6 patients had high risk factors and were referred for adjuvant chemoradiation therapy. Among these 6 cases 4(12.9%) had isolated positive vaginal margin, two (6.4) had both positive margin and pelvic nodal metastasis.

**Table 3:** Intra-operative findings and complications among those LACC who underwent radical hysterectomy in Ethiopia, 2017-2022(n = 31).

Variable	Category	n	%
Parametrial involvement among operated(n = 31)	Yes	2	6.4
Nodal metastasis	Pelvic nodal positive	2	6.4
	Vaginal margin positive	4	12.9
Intra-operative complications	Overall complications	5	16.1
	Bowel injury	2	6.4
	Vascular injury	1	3.2
	Hemorrhage requiring blood transfusion	3	9.7
Postoperative complications	Overall	7	22.6
	PTE	1	3.2
	Wound infection	2	6.4
	DVT	4	12.9

## 4. Discussion

In this study, one-third (31/98, 31.6%) of cervical cancer patients with locally advanced disease were operable following the neoadjuvant chemotherapy (NACT) and had radical hysterectomy. One-fifth (6/31,19.4%) of those who had radical hysterectomy were sent for chemoradiation after the pathology report documented margin positivity with a third (2/6,33.3%) of them having pelvic nodal metastasis.

Although chemoradiation remains the standard treatment for locally advanced cervical cancer, the effectiveness of neoadjuvant chemotherapy plus radical hysterectomy as alternative treatment for such disease has been examined in multiple clinical trials (most of them conducted in high-income settings) [7]. Findings of these clinical trials show that NACT plus hysterectomy has less risk of death (OS) compared to radiotherapy alone [8–10]. However, results of a Cochrane review indicate that there is inadequate evidence to support the survival benefits of hysterectomy with chemotherapy for locally advanced cervical cancer (NACT) [11].

The finding of 31.6% operability among cervical cancer patients with locally advanced disease who received NACT in our study is lower than a 72.1% (31/43) feasible radical surgery achieved following a NACT for locally advanced disease in a Turkish study [12] and 73.3% (11/16) operability rate reported in a recent study from Nigeria [13]. This large difference in the success rate of feasible radical surgery between that found in our study and these previous reports could be explained by the fact that 13 patients disappeared shorty after being assigned to receive chemotherapy with unknown response. Moreover, more than a quarter of patients (25.5%) enrolled for NACT in our study were in Stage-IIB, which is higher compared to that of 9.3% (4/43) in the Turkish study and none (0%) in the Nigerian study were at in similar stage. The higher the disease stage, one would expect the lower the response rate.

Although the period of survival analysis was relatively short (24 months), in our study, radical surgery (radical hysterectomy) was achieved in more than a third of patients who had chemotherapy. The recurrence rate found in our study (3%) is lower than a recurrence rate of 10 to 20% reported in the literature [14]. Nodal metastasis was found in only 2 patients (all pelvic lymph node metastasis) in the present study. Most disease recurrences (83%) occur within 2 years of treatment with poor prognosis [15]. In the present study, patients were followed up to 2 years post-treatment and there was no death reported.

Strengths of our study include being among the first studies to present evidence on NACT plus radical surgery from a low-income setting and larger sample size compared to those previous studies. The main limitations are retrospective study design and lack of comparative analysis with the standard management (chemoradiation). Though small, patients drop out from follow-up, is the other limitation of our study.

In summary, our study supports utilization of neoadjuvant chemotherapy plus radical surgery for locally advanced cervical cancer, where radiotherapy is not readily available, despite a lower feasibility of radical surgery compared to reports from previous studies. It demonstrates that NACT plus radical hysterectomy can be utilized as an alternative to standard chemoradiation in a low-resource setting, such as ours, where disease advancement while waiting for radiation is a common scenario for majority of patients with locally advanced disease. This has relevance to the practice of oncologic care for cervical cancer patients across the region of Sub-Saharan, where radiotherapy is either not available at all or it is not accessible to patients in the needed time due to long waiting list of patients, for example a single radiotherapy center had been serving the whole population(115million) of a country until recently, before three more radiotherapy centers were introduced outside the capital Addis Ababa over the last two years. We recommend further comparative analytic studies.
